# Characteristics and Applications of Sugar Cane Bagasse Ash Waste in Cementitious Materials

**DOI:** 10.3390/ma12010039

**Published:** 2018-12-22

**Authors:** Qing Xu, Tao Ji, San-Ji Gao, Zhengxian Yang, Nengsen Wu

**Affiliations:** 1 College of Transportation and Civil Engineering, Fujian Agriculture and Forestry University, Fuzhou 350116, China; xuqing77@sina.com; 2 College of Civil Engineering, Fuzhou University, Fuzhou 350116, China; jt72@fzu.edu.cn; 3 National Engineering Research Center for Sugarcane, Fujian Agricultural and Forestry University, Fuzhou 350002, China; gaosanji@yahoo.com; 4 Department of Civil & Environmental Engineering, Washington State University, Pullman, WA 99164-2910, USA

**Keywords:** sugar cane bagasse ash (SCBA), pozzolanic activity, agricultural waste, cementitious materials, concrete

## Abstract

Sugar cane bagasse ash (SCBA) is an abundant byproduct of the sugar and ethanol industry. SCBA is generally used as a fertilizer or is disposed of in landfills, which has led to intensified environmental concerns. In recent years, SCBA research has mainly been focused on utilization in construction materials due to the abundance and pozzolanic characteristics of SCBA. In this paper, a comprehensive review of the state-of-the-art morphology, physical properties, chemical composition, and mineralogical composition of SCBA is presented. Studies indicate that SCBA is a potentially promising construction material. The applications of SCBA as a pozzolanic material, a new source for preparing alkali-activated binders, aggregates, and fillers in construction materials, are summarized. The impacts of SCBA on fresh and hardened concrete properties are highlighted, including the physical properties, mechanical strength, microstructure, and durability. Key factors that govern pozzolanic activity are discussed in detail, including calcination and recalcination temperatures, and durations, fineness, loss on ignition (LOI), and crystal silicon dioxide. Finally, further research on the optimal and broad utilization of SCBA in construction materials is recommended.

## 1. Introduction

Concrete is the most widely used, manmade material in the world. Portland cement is normally an essential binder ingredient used in concrete. Cement production consumes a considerable amount of raw material and energy, and releases a large quantity of CO_2_. To reduce carbon emissions, attempts have been made to find substitutes for cement to minimize the environmental impact of the concrete industry.

With rapid industrial and agricultural development, large quantities of industrial and agricultural waste have been generated. Disposal of these wastes is a serious environmental problem, as most final wastes go to landfills, which not only reduces useful land area but also pollutes the environment. Industrial byproducts, such as coal fly ash, silica fume, and blast furnace slag, have been successfully used in cementitious materials and have achieved sufficient social and environmental benefits. Currently, agricultural and forestry residues are mainly used as biomass fuel. The resulting bottom ash or fume ash is the final waste, which has aroused wide research interest. It has been found that many different kinds of biomass ash can be used as supplementary cementitious materials, such as rice husk ash [1], palm oil fuel ash [2], elephant grass ash [3], sugar cane bagasse ash [4], corn cob ash [5], wood waste ash [6], bamboo stem ash [7], cattle manure ash [8], and paper mill ash [9]. Previous studies found that incorporating biomass ash into cementitious materials could maintain or even improve the mechanical performance of the cementitious materials. Additionally, the incorporation of biomass ash into cementitious materials can help to reduce the greenhouse gases produced in cement production, lower the costs of construction materials, alleviate waste disposal pressure, and prevent soil and air pollution.

Sugar cane is a kind of tropical and subtropical crop and is the main sugar crop worldwide. Global sugar crop acreage is approximately 31.3 million hectares, among which sugar cane accounts for approximately 70%. The world's top three sugar-producing countries are Brazil, India, and China, which accounted for 20.57%, 16.91%, and 6.31% of the global production in 2016, respectively. Recently, sugar cane acreage reached approximately 1.23 million hectares in China, and production was approximately 100 million tons in 2017/2018.

**Figure 1:**
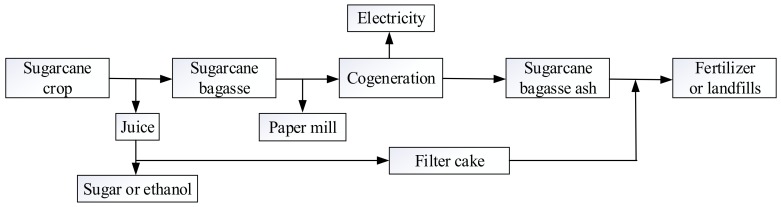
The generation process of SCBA in a sugar mill.

Sugar cane is typically used to produce sugar and ethanol. Figure 1 shows the whole production chain in the sugar mill. After the extraction of sugar juice from sugar cane, sugar cane bagasse is produced, which is approximately 50% of the sugar cane quality. Bagasse is commonly used as a fuel in cogeneration to produce steam and generate electricity. In this process, sugar cane bagasse ash (SCBA) remains as the final waste in the sugar production chain. Each ton of burnt bagasse may generate 25–40 kg of bagasse ash [10] and, subsequently, a considerable amount of SCBA could be generated. With the increasing demand for more sugar and ethanol production in recent years, SCBA outputs have substantially increased. In China alone, there may be 1.25–2 million tons of SCBA produced each year. After mixing with sugar cane filter cake or vinasse, SCBA is commonly used as a fertilizer in sugar cane plantations in Brazil [10] and China. However, SCBA is generally disposed of in landfills in India [11]. 

Sales and Lima [10] found SCBA did not have enough mineral nutrients to be used as a fertilizer, and the solubilization test revealed that aluminum, chromium, plumb and total phenol exceeded the level allowed by the Brazilian NBR 10006 standard. Therefore, using bagasse ash as fertilizer has a low nutritional value and heavy metals may penetrate into the ground and pollute the soil and groundwater, thereby triggering potentially serious social and health problems. In this context, SCBA is not recommended as a fertilizer. There is a great need to seek new solutions for disposal of SCBA produced in sugar-ethanol plants.

Researchers have been exploring new SCBA utilizations. These new SCBA uses include production of glass-ceramic material [12], geopolymers [13], raw material for ceramic [14], phillipsite zeolite synthesis [15], Fe_2_O_3_-SiO_2_ nanocomposite to remove Cr(VI) [16], sodium waterglass [17], silica aerogels [18], and mesoporous silica as a catalyst [19]. It should be emphasized that considerable SCBA research has been focused on utilization in cementitious materials. In 1998, Hernández et al. [20] found that as a byproduct of sugar milling, sugar cane straw ash showed good pozzolanic activity, which was comparable to rice husk ash. Since then, SCBA has been widely studied as a pozzolanic material. The positive effects of bagasse ash on concrete performance have been studied. SCBA incorporation into concrete can improve the mechanical properties of concrete at certain replacement levels [21], decrease hydration heat [22,23], improve concrete durability [24,25,26], and intensify the interface between the cementitious matrix and the aggregate [27]. These studies proved the feasibility of using SCBA in cementitious materials and demonstrated the potential engineering value of SCBA. In addition, the utilization of SCBA in cementitious material is profoundly significant in view of waste management, environmental protection, cost reduction and natural resource conservation.

## 2. SCBA Characterization 

The physical properties and compositions of SCBA vary with many factors, such as sugar cane varieties, growth, combustion temperature, combustion duration, purity of bagasse, bagasse ash collection location, cooling type, boiler equipment, bagasse ash collection methods and ash fineness [28]. For example, bagasse ash collected from the bottom of the boiler may be coarser and contain irregular particles, and the bagasse ash collected through a filtration system contains less carbon [29].

### 2.1. Morphology and Physical Properties

#### 2.1.1. Macroscopic Morphology and Physical Properties

The color of power plant-generated SCBA is dark black to light black. Dark black indicates a higher carbon content, mainly due to incomplete combustion [28]. Owing to crystallization and decomposition at high temperatures, SCBA appears gray above 800 °C recalcination temperature and appears white above 900 °C [30].

**Figure 2:**
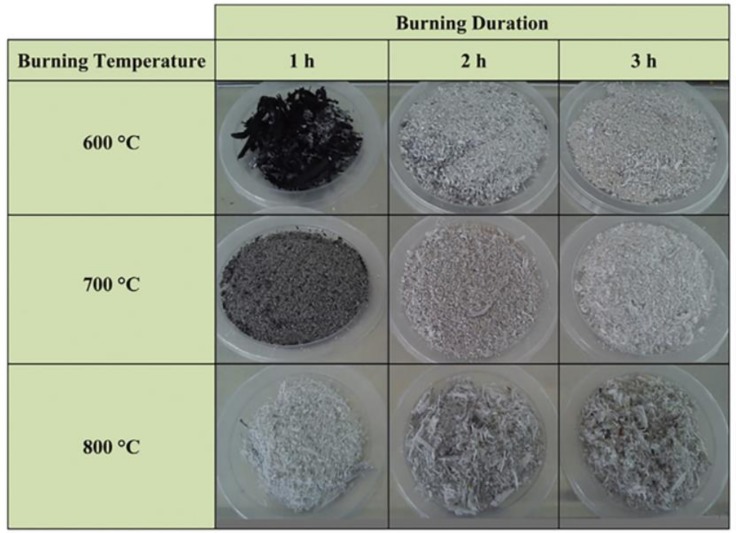
Appearance of SCBA with different calcination temperatures and durations. Reproduced with permission from [31].

Embong [31] calcinated sugar cane bagasse at different temperatures and for different durations. Figure 2 shows obtained SCBA with different micromorphology, color, and fineness. As the calcination temperature and duration increases, the ash gradually changes from char to white ash. SCBA from power plants is produced under uncontrolled calcinated temperatures and durations and, therefore, the SCBA is a mix of different micromorphology and color particles.

Due to the presence of lightweight, porous, fibrous carbon particles, the specific surface area (145 m^2^/kg) and specific gravity (1.91) of raw SCBA are relatively low [30]. The specific surface area and specific gravity varies with burning temperature, as the SCBA composition and morphology changes with the burning temperature [30].

#### 2.1.2. Micromorphology

**Figure 3:**
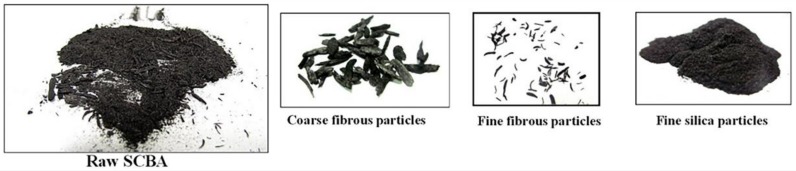
Morphology of raw SCBA from power plants. Reproduced with permission from [32].

As illustrated in Figure 3, SCBA collected from power plants have silica-rich, fine, thoroughly burnt particles and carbon-rich, fibrous, unburnt particles [32]. Four shapes are found in fine particles: spherical, prismatic, fibrous and irregular. Prismatic particles and irregular particles are rich in silicon. The prismatic structure in the white particles without carbon particles reveals crystallization that may lower the pozzolanic activity of SCBA [30]. Spherical particles are mainly formed by melting at high temperature and contain Mg, P, K, Si, Na, Fe and other minor elements [30,33].

Coarse, fibrous particles are easy to identify because the particles are relatively large. Fibrous, unburnt particles are generally very rough and have a unique microstructure, and the main component is unburnt carbon rather than silicon [30]. The epidermal layer with a dumbbell shape is randomly distributed on the face of the unburnt carbon layer. The major elements of the dumbbell particles are Si, O, and C [30]. The structure in fine, fibrous unburnt particles in the cell wall is different from that in coarse, fibrous unburnt particles. The cell structure with intercellular channels in the cell walls can be observed. More than 80% of the elemental composition was carbon.

### 2.2. Chemical Compositions

Table 1 shows the chemical composition of SCBA in earlier studies. The chemical composition is significantly different among different SCBA samples. However, the samples share the main components of silica, aluminum, and other metal elements. According to the ASTM C618-08a specifications, natural pozzolans with a ratio of the SiO_2_ + Al_2_O_3_ + Fe_2_O_3_ sum to the total mass above 50% may be categorized as class C pozzolans, and as class F pozzolans when that ratio is above 70%. Based on this criterion, most SCBA samples in Table 1 could be judged as class F pozzolans.

### 2.3. Mineralogical Compositions

The silica in SCBA presents in amorphous and crystalline forms (quartz and cristobalite) [4]. Reactive amorphous silica of sugar cane comes from groundwater. Sugar cane absorbs silicic acid from groundwater and then polymerizes into amorphous silica in sugar cane cells [37]. When sugar cane bagasse is burnt in a thermal power plant boiler as a fuel, reactive amorphous silica is produced and remains in the final SCBA residue [22].

The crystalline silica is generally considered to be an inert compound [38]. Crystalline silica generally comes from two sources: the uncontrolled incineration process and the sand in the soil. Crystalline silica is generally associated with high incineration temperatures (>800 °C) and long incineration durations [39,40]. In addition, the crystalline silica may also come from the sand in the soil. The sand adheres to the sugar cane when the sugar cane is harvested, squeezed and incinerated, and is eventually brought to the SCBA. Although the amount of the sand that adheres to the sugar cane is small (approximately 2%), after boiler incineration the mass ratio of the sand to the SCBA as an impurity significantly increases to more than 50% [39]. When the SCBA has a relatively high silica content in the form of quartz, it can still be used in building materials as a sand replacement or filler [10].

**Figure 4:**
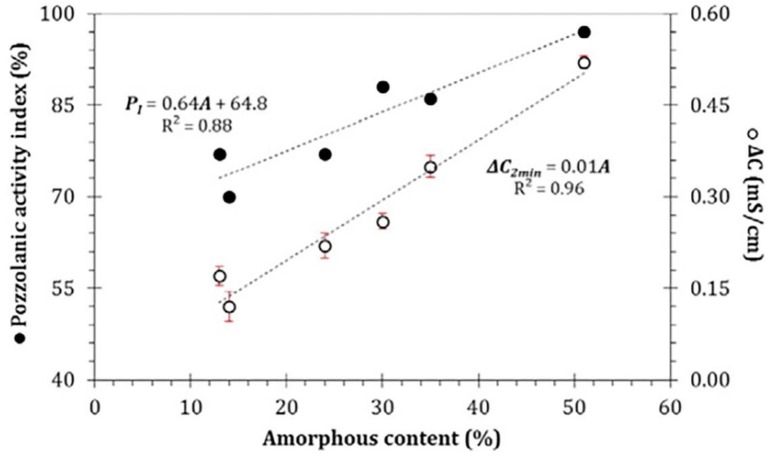
Relationships among conductivity data variation (*ΔC*), pozzolanic activity index (*P_I_*) and total amorphous phase content (*A*). Reproduced with permission from [38].

Due to the high amount of amorphous or partially crystalline silica and amorphous alumina (essential components of the pozzolanic reaction with calcium hydroxide), SCBA can be used as a pozzolanic material [41]. Figure 4 shows the relationships among the conductivity data variation (*ΔC*), pozzolanic activity index (*P_I_*) and total amorphous content (*A*). With an increase in the SCBA amorphous content, there was a systematic increase in both *ΔC* and *P_I_*, and direct linear relationships were observed with correlation coefficients (R^2^) of 0.96 and 0.88, respectively.

SCBA from different sources must be individually analyzed for its chemical composition and mineralogical compositions to determine the optimal use of the SCBA [42].

## 3. SCBA in Cementitious Materials

Recently, very promising results from studies on the multiple SCBA applications in cementitious materials have been reported. SCBA applications in cementitious materials can be divided into the following categories.

### 3.1. SCBA as Pozzolanic Materials 

Due to the relatively high amorphous silica content, SCBA has been widely studied as a pozzolanic material to partially replace cement. This use of SCBA is the mainstream of the present studies on SCBA.

#### 3.1.1. SCBA as A Pozzolanic Material in Concrete

SCBA has been widely incorporated into concrete as a pozzolanic material, such as conventional concrete [32,34], high-performance concrete [43], self-compacting concrete [44,45], and recycled aggregate concrete [46]. These applications are considered to be valuable uses of SCBA.

#### 3.1.2. SCBA as A Soil Stabilization Material

In some cases, soil properties may not meet the required engineering specifications. Soil stabilization is the main method used for improving soil properties. Osinubi et al. [47] investigated the possibility of using SCBA to stabilize lateritic soil and found that SCBA cannot be used as a stabilizer alone and must be used with adequate cement stabilization. Jamsawang et al. [48] partially replaced ordinary Portland cement (OPC) with SCBA as a cheap pozzolanic material and mixed SCBA with soft clay to produce SCBA-admixed soft clay. The test results showed that the replacement of OPC with SCBA could achieve the same strengthening effect when OPC is used alone. The optimal amount for improving the strength of the soft clay is considered to be 20% replacement of OPC with SCBA. 

To explore the potential of SCBA as a subgrade material, Khan et al. [49] mixed cement with SCBA in different proportions. The optimal content is 9% cement–SCBA mix, as the highest maximum dry density (727.8 kg/m^3^) and compressive strength value (6.46 tons/m^2^) were observed. The highest California bearing ratio (CBR) values were 7.3% for the 4-day cured sample and 23% for 40-day cured sample. 

#### 3.1.3. SCBA as A Pozzolanic Material in Other Construction Materials

In addition to utilization in concrete, SCBA can be used in unburnt bricks and paver blocks [11]. Deepika et al. [11] used raw SCBA, OPC 53 grade cement, crusher sand, and river sand to produce unburnt brick materials. A total of 46 kg of raw SCBA and 12 kg of cement were adopted for 100 kg of mix, and the water-to-binder ratio was 0.15. Except for water absorption (which is still above the permissible limit specified in the standard), the properties of SCBA-based unburnt bricks were better than those of fly ash bricks. In particular, no efflorescence was observed [11]. Deepika et al. also produced precast concrete paver blocks at 10% and 20% replacement of cement with SCBA. Marginal decrease in compressive strength was observed. However, the strengths were still well above the minimum requirement (35 MPa). In terms of water permeability and water sorptivity, SCBA blended paver block specimens performed better than control specimens because of the pozzolanic performance [11]. 

### 3.2. SCBA as Alternatives to Other Ingredients in Construction Materials

Considerable research has considered SCBA as a pozzolanic material. Notably, not all SCBA samples possess good pozzolanic reactivity. Some SCBA even possesses poor pozzolanic activity. Even in this case, because of unique properties, SCBA can still be used in building materials [10,50]. This is another way to utilize SCBA, which could reduce the use of naturally mined materials.

#### 3.2.1. Applications in Alkali-Activated Systems

Alkali-activated systems are considered to be promising green binding materials with good mechanical properties and durability [51]. SCBA has been demonstrated as a potential source for preparing alkali-activated binders. Moraes et al. [36] successfully used SCBA in blast furnace slag (BFS)-based alkali-activated binders. Due to the dissolution of silicate anions from the SCBA, the compressive strength of SCSA/BFS samples is higher than 50 MPa after 90 days, which is similar to those obtained for BFS mortars. In addition, SCSA/BFS samples can be activated with sodium hydroxide, while not sodium silicate, which significantly decreases the cost of the binder [36].

#### 3.2.2. SCBA as A Filler in Concrete

When silica polymorph phases change to α-quartz, the SCBA pozzolanic activity is largely reduced [42]. Despite an apparent lack of pozzolanic activity, SCBA still has a good filler effect in concrete [52]. Due to the filler effect, 20% SCBA by weight of the cement can produce 40 MPa concretes with superplasticizer, while, in the absence of superplasticizer, 5% is the optimal replacement level for the compressive strength, acid resistance, and drying shrinkage [52]. 

Additionally, SCBA can replace natural clay and can be used as filler in clay bricks [53]. However, the decrease of mechanical strength and the increase of water absorption limit the incorporation of SCBA into clay bricks. Low concentrations (less than 10 wt%) of SCBA into clay brick body are recommended [53].

#### 3.2.3. SCBA as A Partial Substitute for Aggregates in Concrete

Because of the crystalline structure, SCBA can replace both Portland cement and fine aggregate [10,54,55]. SCBA with a high silicon content has similar physical properties to natural sand. Compared to the reference samples, replacing 20% and 30% of sand with SCBA would improve the compressive strength of the mortars [10]. There is evidence showing that SCBA could be used as a substitute for fine aggregates [10,54]. Lura et al. [56] produced biolightweight aggregates with SCBA and investigated the pore structure, water absorption and desorption behavior of the new aggregates. The results showed that the biolightweight aggregate could be used as an internal curing agent in high-performance cementitious systems.

## 4. SCBA Impacts on Concrete Properties

Table 2 shows the detailed impacts (most are positive) of SCBA on concrete properties. SCBA can modify the particle size distribution of the cementitious matrix, increase concrete rheology, and improve the chemical and physical properties of the cementitious material [27]. These changes were caused by SCBA pozzolanic reactivity and the resulting refinement in pore size distribution [39].

### 4.1. Effects on Concrete Physical Properties 

Due to thermal stress and temperature gradient, a high rate of heat evolution in concrete leads to early age thermal cracking [32]. The total heat and peak heat rate of the SCBA-blended concrete are lower than the total heat and peak heat rate of the control group [32,34]. Compared to the control concrete, the peak temperature of the SCBA concrete could be reduced by 4–11 °C, and the time to reach the peak temperature was delayed 1–3 h, depending on the replacement level [34]. The C_3_A, C_3_S, and gypsum in the cement remarkably influence cement heat evolution. The replacement of cement by SCBA would decrease the contents of C_3_A and C_3_S, leading to a reduction in hydration heat [32]. Therefore, SCBA can be used in concrete with low hydration heat requirements, such as mass concrete work.

For the SCBA effect on concrete drying shrinkage, Bahurudeen et al. found no significant length change between the control and SCBA concretes (0, 5, 15, and 25 wt% OPC) [32]. Arif et al. replaced sand with 0, 5, and 10% SCBA and found that concrete with 5% SCBA replacement had the lowest shrinking strain of 958 microstrain, which is much lower than the control concrete with 1660 microstrain [52].

Bahurudeen et al. found that with an increased SCBA replacement level, the surface resistivity of the SCBA concrete increased at 28 and 56 days, especially for the 15% and 25% SCBA replacement level specimens. This result proved an enhancement in the pore structure and hence a reduction in both the corrosion risk and concrete permeability [32].

Partial replacement of cement by SCBA in concrete would prolong the initial and final setting times of the blended concrete [21,57,58]. The setting time delay is mainly due to the following factors [58]: higher amount of mixing water [66], reduction in cement content [67], and presence of SCBA particle layers around the anhydrous cement particles that would decrease the cement hydration [58].

Some researchers reported that a higher SCBA percentage increased the water requirement for normal consistency of cement paste, and the concrete water demand increased when the SCBA replacement level increased [21,57,58,61]. This result was possibly due to the higher specific surface area of the SCBA particles relative to cement, caused by the irregular and porous surfaces of the SCBA particles [28]. In contrast, other researchers found that the workability of concrete with SCBA increased linearly when the increased SCBA content was under 30% [56,60], which can be attributed to the SCBA glassy texture and low value of LOI. If the workability (slump value) of concrete is kept constant, the SCBA concrete would require less mixing water, which could further increase the compressive strength.

The soundness of paste samples with SCBA was better than the control sample without SCBA [32]. The opposite case was also reported. The paste specimens with SCBA showed a small expansion, but the expansion was still less than the permissible limit of soundness [30].

### 4.2. Effects on Concrete Mechanical and Microstructural Properties

In general, SCBA concrete shows an excellent performance when SCBA partially replaces cement. There are two main reasons: (a) the high amorphous silica content in SCBA triggers pozzolanic reactivity and (b) the ultrafine particle sizes of the SCBA significantly improve the microstructure, leading to a high early strength [59,60,68,69].

Many researchers found that the compressive strength of concrete with SCBA increased first and then decreased when the replacement ratio was increased. The optimal replacement ratio for compressive strength was different in previous research, for example, 15% [65], 20% [21,63], and 25% [32,34]. Rerkpiboon et al. found that concrete with 50% ground SCBA reached at least 90% of the control concretes’ compressive strength at the age of 28 days [63].

**Figure 5:**
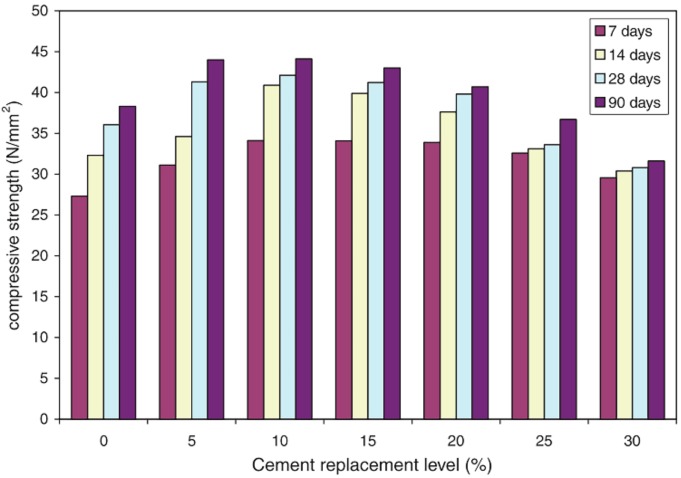
Compressive strength of SCBA-blended concretes. Reproduced with permission from [21].

Figure 5 shows the early compressive strength development of concrete with and without SCBA. The reasons for the early compressive strength development of SCBA concrete may be the high SCBA fineness, which fills the voids between the cement and aggregates [21]. At later ages, the pozzolanic reaction between reactive silica in SCBA and calcium hydroxide generated from the hydration process of cement formed additional calcium silicate hydrate (C–S–H), which would improve the interface bond between pastes and aggregates [21,22,63,64].

A different finding was reported by Tantawy et al., who found that the compressive strength decreased with SCBA incorporation at all ages [58]. This difference could be caused by the SCBA particles covering the anhydrous cement particles at early ages and therefore reducing the rate of cement hydration. At later ages, SCBA reacted with portlandite in the pastes and generated additional calcium silicate hydrates, which enhanced the compressive strength. However, the compressive strength of SCBA concrete is still less than that of the control concrete at all ages, which may be due to the decrease in OPC content [58].

There is no consensus on the SCBA effects on the concrete splitting tensile strength. Ganesan et al. found that the splitting tensile strength of the SCBA concrete increased first and then decreased with increasing replacement level, and the optimal replacement level was 20% [21,62].

Arif et al. [52] studied the effects of SCBA on the flexural strength of concrete. He substituted the sand with SCBA in both stage-one and stage-two concrete with the same water/binder (W/B) ratio of 0.45 by cement weight. There was no superplasticizer in stage-one, so the specified slump varied. For stage-two, a superplasticizer was added to maintain the same workability. The flexural strength of the stage-one concretes increased with increasing SCBA content and age. However, the flexural strength of the stage-two concretes did not greatly differ from the flexural strength of the control concrete [52].

Rerkpiboon et al. found that concrete with up to 50% SCBA incorporation had an elasticity modulus similar to that of the control concrete [63]. However, Srinivasan et al. found that with increasing SCBA replacement level, the elasticity modulus decreased [60]. The researchers thought there were two reasons for this result: (i) improper SCBA dispersion formed weaker zones, causing the elasticity modulus decrease of the concrete, and (ii) the amount of SCBA exceeded the amount required to react with Ca(OH)_2_. The excess amount of unreacted silica caused a decrease in strength and elasticity modulus [60].

Some research found the concrete porosity was higher with different SCBA replacement levels than that of control concrete without SCBA, which means that the more SCBA that was used in the concrete, the greater the porosity of the concrete [58,64].

Incorporation of a large amount of SCBA decreases the amount of OPC used and increases the mixing water amount, which leads to a high concrete total porosity [58]. The fine SCBA particles segment the large pores and generate nucleation sites for hydration product precipitation [64]. The SCBA refined the pore structure and reduced Ca(OH)_2_ from the hydration reactivity in the paste, although the porosity increased. With a prolonged curing time, the porosities of the concretes declined because of the continuous hydration of cementitious materials [58].

The narrow thickness of the interfacial transition zone (ITZ) leads to higher compression because the bond between the cement paste and aggregate becomes stronger. Due to fineness and pozzolanic reactivity, SCBA refined the pore sizes and densified the interfacial zone, and the ITZ thickness was obviously reduced at a replacement level of less than 30%. This result can clearly be confirmed by the improved compressive strength results [59]. Incorporating SCBA into concrete narrows the ITZ thickness, and increases the elasticity modulus and hardness of the ITZ [27].

### 4.3. Effects on Concrete Durability

Many studies have found that SCBA could increase concrete durability [24,63,70]. Bahurudeen et al. found that concrete blended with SCBA had a superior performance in oxygen and Torrent air permeability after 28 days of curing, and the permeability performance further improved during 56 days of curing as a result of pozzolanic reactions [32].

Bahurudeen et al. also found that the water penetration of the SCBA-blended concrete significantly reduced after both 28 and 56 days of curing [32]. Ganesan et al. found that the water absorption percentage increased in the presence of SCBA during 28 days of curing, which is possibly due to the fact that SCBA is hygroscopic in nature and finer than OPC. However, the water absorption percentage decreased obviously (50%) after 90 days of curing. This result is due to the addition of SCBA that decreased permeable voids and the gradual closing of pores [21]. Evidently, the use of SCBA significantly improves the resistance of concrete to water penetration.

Partial replacement of cement with SCBA could significantly reduce the chloride permeability and chloride diffusion of concrete. The charge passed through the SCBA concrete was significantly less than that of the control concrete mix [21,32,63]. The reduction in chloride permeability may be attributed to the finer SCBA particles. Additional C–S–H gel from pozzolanic reactions precipitated into the oversized pores. Finer SCBA particles may cause oversized pore breakup or, in other words, lower pore connectivity and pore solution conductivity. Thus, the chloride penetration values decreased [32,64]. Notably, the possible electrical conductance by the SCBA and/or the superplasticizer may lead to this misleading result [71].

Joshaghani evaluated the sulfate resistance of mortars containing SCBA by concrete compressive strength loss and weight loss for six months. He found that after storage in sulfate, the compressive strength loss and weight loss were less in the mortar specimens containing SCBA than those of the control specimens at all ages under study. Twenty percent SBCA replacement showed the least compressive strength loss and weight loss. The SCBA replacement decreased the C_3_A and Ca(OH)_2_ and hence reduced the possibility of transformation from gypsum to ettringite, which is a sulfate attack risk [24].

Gar et al. [65] found the compressive strength of concrete dropped continuously at elevated temperature, but the SCBA could partially counteract the downward trend. The SCBA incorporation decreased the flexural strength of the concrete at elevated temperatures, and the decrease ranged from 20% to 40%. The 10% SCBA cement substitution appeared to have a minimum decrease in flexural strength.

Katare and Madurwar summarized the optimal SCBA replacement range for various properties of concrete according to the earlier research results, as shown in Table 3. The researchers found that 25% was the optimal replacement level, at which the best overall performance of SCBA-blended concretes can be achieved [28].

## 5. Strategies for Improving SCBA Pozzolanic Activity

In most cases, supplementary cementitious materials cannot be used directly as pozzolanic material. They need instead to be processed to meet the certain standards for pozzolanic materials. Although SCBA has been identified as an agricultural-based pozzolanic material, SCBA cannot be directly used in concrete because of poor pozzolanic activity. The pozzolanic activity of SCBA is mainly caused by crystalline silica and impurities such as unburnt carbon [20]. The relatively low amorphous silica content (typically less than 50% silica) [29] makes SCBA less popular than rice husk ash. For large-scale SCBA applications, various processing methods have been used in previous studies to improve the pozzolanic activity of SCBA.

### 5.1. Controlling the Calcination and Recalcination Temperature of SCBA

Calcination temperature significantly influences the pozzolanic activity of supplementary cementitious materials, such as rice husk ash [72] and metakaolin [73]. Calcination temperature also has a similar effect on SCBA [40], as the mineralogical composition and morphology of SCBA are significantly influenced by calcination temperature and duration [40,74].

High SCBA pozzolanic activity could be obtained by controlling calcination temperature and duration, whereby most silica is kept in a noncrystalline form [74]. Different boiler/furnace systems usually run on different combustion temperatures ranging from 550 to 1000 °C [30,42]. In the case of 50% bagasse humidity, the flame temperature of the burning bagasse may vary from 850 to 920 °C and if the bagasse humidity is less than 35%, the flame temperature may reach 1000 °C [10]. A high combustion temperature within the boiler can result in silica crystallization, which eventually leads to poor SCBA pozzolanic activity [39].

Incomplete combustion would result in high carbon content in SCBA. Calcining bagasse under low temperature (600 °C) for a short time (1 h) would generate a char-like ash, which contains cristobalite SiO_2_ and graphite (carbon). Prolonged heating is necessary for the complete combustion of sugar cane bagasse into white ash [31].

**Figure 6:**
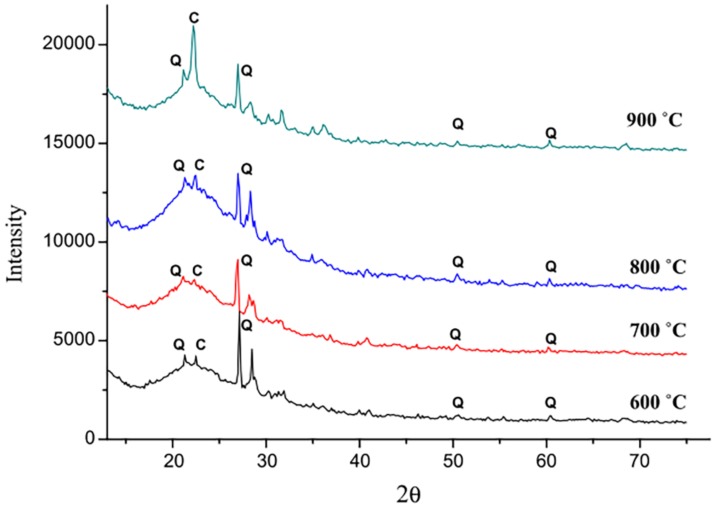
X-ray diffraction pattern of the burnt SCBA samples (Q: quartz, C: cristobalite). Reproduced with permission from [30].

The power plant SCBA is normally black with a higher carbon content [28]. Bahurudeen et al. [30] recalcined plant-obtained SCBA at different temperatures, including 600, 700, 800 and 900 °C for 90 min at each temperature. The recalcined SCBA was then chilled in the air. Table 4 presents the results of the strength activity index (SAI) of four samples calcined under different temperatures. The SCBA pozzolanic activity recalcined at 800 °C is stronger than that of the raw SCBA. The sample calcined at 700 °C had the highest pozzolanic activity. The gradual transformation from amorphous silica to cristobalite above 700 °C is clearly shown in Figure 6, resulting in a pozzolanic activity reduction.

In the recalcination process, besides the calcination temperature and duration, the cooling method is another key factor in determining the color and chemical compositions of SCBA. The SCBA can be chilled at room temperature [30]. Cooling the SCBA slowly in the oven [74,75] would lead to different properties. Unfortunately, some of the research did not provide the cooling methods in detail [76]. Until now, the effects of the two different cooling methods on the SCBA properties have not been found.

### 5.2. Controlling SCBA Fineness

**Figure 7:**
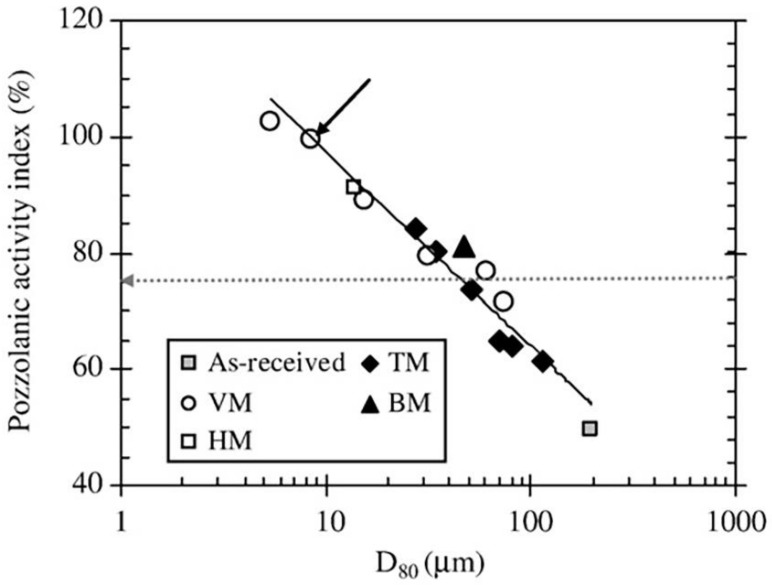
Relationship between pozzolanic activity index and D_80_. Reproduced with permission from [39].

The auxiliary cementitious material activity is affected by the particle fineness. For relatively coarse particles (D50 ≈ 30 µm), SCBA is more like an inert mineral admixture with poor pozzolanic activity [4]. A number of studies have shown that raw SCBA pozzolanic activity was weak, and the raw SCBA must be ground to a greater level of fineness (300–320 m^2^/kg) for higher pozzolanic activity and workability [34,39,77]. The pozzolanic activity index of SCBA can be increased from 50% to 100% by an increase in the SCBA fineness, as illustrated in Figure 7 [39]. Only through ball grinding is it possible to convert SCBA from agricultural waste into mineral admixtures.

**Figure 8:**
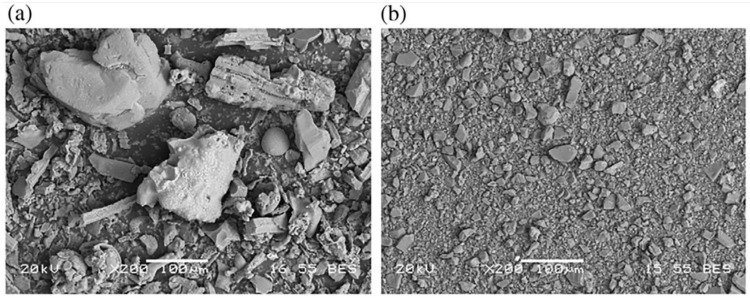
Micromorphology of SCBA produced after 8 min (**a**) and 240 min (**b**) grinding [39].

Grinding, which is commonly used to reduce the negative impact of crystalline silica, improves SCBA homogeneity and increases pozzolanic activity, and is accompanied by increases in specific surface area and the introduction of defects and reaction cores on the SCBA surfaces [4]. Figure 8 shows the micromorphology of the SCBA ground for different amounts of time. The SCBA particle size clearly decreased with increased duration of grounding.

However, when the loss on ignition (LOI) of SCBA is relatively high (high carbon particle content), grinding alone could not significantly improve SCBA pozzolanic activity. In this case, grinding must cooperate with other processing methods [30].

To reach the pozzolanic activity index lowest standard of 75% (D80 = 60 µm), the energy required for SCBA would be approximately 42 kWh/t. To reach the pozzolanic activity index of 100% (D80 = 9 µm), the energy required would be in the order of 250 kWh/t [39]. To determine the optimal grinding process, energy efficiency should be considered. Notably, in any case, SCBA energy consumption is less than that of cement of the same weight.

### 5.3. Lowering the Loss of Ignition (LOI) of SCBA

In addition to the main components of silica, alumina and iron oxide, bagasse ash also contains carbon (carbonate and unburnt organic carbon) and water; both are expressed as LOI [42]. In ASTM C618-08a, the LOI required for nature pozzolans is less than 6% for class C and less than 10% for class N. Twelve percent LOI is approved for class F under certain circumstances.

The high LOI limits the potential use of biomass-derived ash as a concrete admixture and in construction applications [56]. In 1998, Hernández et al. [20] found that sugar cane straw ash as a byproduct of sugar milling showed good pozzolanic activity. However, raw SCBA was not an active pozzolan due to the presence of unburnt material and carbon.

High LOI makes SCBA appear dark black [74]. High LOI also leads to a greater water demand for the same workability, which means reducing LOI could improve workability [30]. In 2009, Chusilp et al. found that the high SCBA LOI obviously decreased the compressive strength of mortar at an early age but had little effect on the compressive strength at a later stage [68]. However, if the LOI is less than 10%, the pozzolanic activity would improve and could partially replace cement for use in cementitious materials [68]. In addition to the impact on pozzolanic activity, LOI had an adverse effect on the sulfate resistance of mortar [68].

Low LOI can be achieved in the following ways: (1) Floatation—since there is a very small amount of soluble material in the bagasse ash, the fibrous unburnt particles could be separated by means of floatation [33]. (2) Recalcination can also be used to reduce LOI, but if the calcination temperature exceeds 550 °C, the LOI reduction is no longer significant [68]. Considering energy consumption and the conversion from amorphous silicon to crystalline silicon, the recalcination temperature should not exceed 600 °C. (3) Using an industrial sieve shaker to screen the carbon particles to decrease LOI, Bahurudeen et al. found that SCBA that passed through a 300 μm sieve left only burn-off particulates rich in silica, and thus the fibrous carbon particles can be removed through a 300 μm sieve [30]. Screened carbon particles could be used as fuel or activated carbon [33]. Screening the carbon particles is an energy-saving screening method to reduce LOI.

### 5.4. Combined Application of Processing Methods

**Figure 9:**
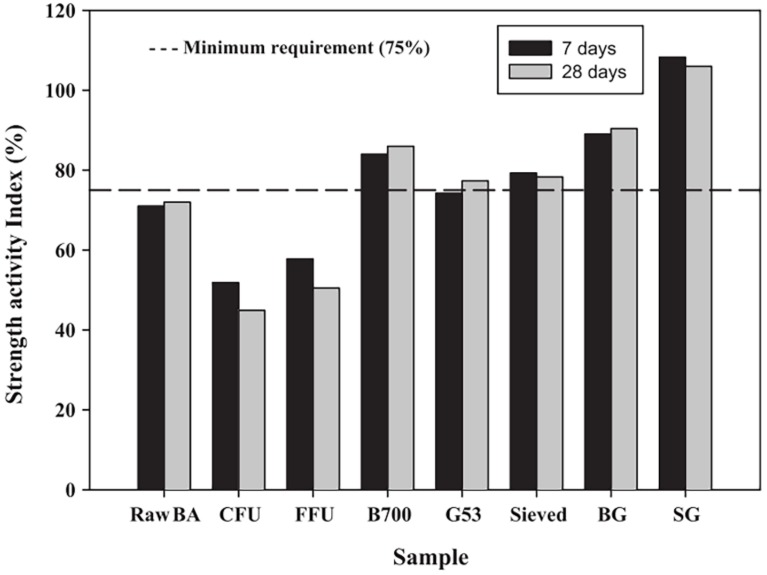
Comparison of SCBA SAI values with different processing methods. Reproduced with permission from [30].

In 2015, Bahurudeen and Santhanam [30] comprehensively evaluated the pozzolanic activity of eight SCBA samples with different processing methods. The eight samples were (1) raw bagasse ash (raw BA); (2) coarse fibrous unburnt (CFU) SCBA (74% LOI); (3) fine fibrous unburnt (FFU) SCBA (72% LOI); (4) bagasse ash burnt to 700 °C (B700); (5) bagasse ash ground to finer than 53 µm (G53); (6) (sieved) bagasse ash sieved through 300 µm sieve; (7) bagasse ash burnt at 700 °C and then ground to cement fineness (BG), i.e., 300 m^2^/kg; and (8) bagasse ash sieved though a 300 µm sieve and then ground to cement fineness (SG). According to the results shown in Figure 9, although there are many methods to improve pozzolanic activity, it is difficult to achieve the desired SAI using only a single method. The integrated application of two or more processing methods is therefore necessary. By decreasing LOI (B700) or increasing fineness (G53, sieved), the pozzolanic activity is slightly improved. Significant improvement in pozzolanic activity can be achieved only by increasing fineness and lowering LOI. Among the eight samples, SG had the highest SAI. The SG processing method requires minimum processing energy.

### 5.5. Other Processing Methods

Crystalline silica is generally considered to be an inert compound [38]. Therefore, the high content of crystalline silica in SCBA would reduce the pozzolanic activity. To reduce the quartz content in SCBA, Cordeiro et al. proposed a two-stage classification grinding circuit to remove quartz-rich waste. The sieved bagasse ash (<212 μm) was finely ground, and the pozzolanic activity of the final SCBA was thus improved. Since the strength of the quartz particles is over 15 times the strength of the bagasse ash particles, the removal of quartz could also reduce ultrafine grinding energy costs [38].

To increase the amorphous silica content in bagasse ash, Embong et al. [31] soaked the bagasse in 0.1 M dilute hydrochloric acid, dried the bagasse in sunshine and calcined at 800 °C for one hour. The amorphous silica content of the obtained SCBA increased to 84%, which led to a higher pozzolanic activity. The obtained SCBA was both ultrafine and chemically stable.

The desired processing methods should attain maximum SCBA pozzolanic activity, while the processing is the simplest and the energy consumption is the lowest, which needs further exploration.

## 6. Conclusions

SCBA is conventionally used as fertilizer or is disposed of in landfills, both of which are not sustainable from the standpoint of environmental and health concerns. Utilization of SCBA in construction materials offers a promising solution for superior recycling and management of SCBA wastes. By consolidating the findings from the existing research, the following conclusions are obtained:

(1) Many studies have reported the successful utilization of SCBA in cementitious material, which improved the short-term mechanical properties and long-term durability of mortar, concrete, and other construction materials. The durability of concrete containing SCBA exposed to severe environments lacks in-depth analyses. The influence of SCBA on reinforced concrete (RC) has rarely been reported.
(2) The heterogeneity of SCBA constrains the large-scale application of SCBA in cementitious materials. Calcination is one of the most important influencing factors on SCBA composition. More attention should be focused on the cogenerator design, calculation control, bagasse drying process, etc., to obtain desirable SCBA pozzolanic activity.
(3) Comparison studies showed that there were both advantages and limitations of different processing methods that affect the SCBA pozzolanic activity. For specific applications, care should be taken in exploring suitable SCBA processing methods for maximum SCBA pozzolanic activity, simplest processing and lowest energy consumption.
(4) Recently, ultrahigh performance concrete (UHPC) has become a research hotspot in civil engineering materials. Rice husk ash has been successfully used in UHPC as both a pozzolanic admixture and an internal curing agent. However, the potential utilization of SCBA in UHPC remains a virgin field well worth being explored.
(5) For the large-scale application of SCBA, further research is needed on technical and environmental aspects, and standardization and governmental policy guidance.

## Figures and Tables

**Table 1:** Chemical composition of SCBA, in wt%.

SCBA												
SiO_2_	Al_2_O_3_	Fe_2_O_3_	CaO	MgO	SO_3_	Na_2_O	K_2_O	P_2_O_5_	LOI	SiO_2_ + Al_2_O_3_ + Fe_2_O_3_	Class	Ref.
76.8	4.4	8	5.4	0.9	0.1	-	-	-	3.3	89.2	F	[34]
67.1	5.7	2.5	2.9	0.5	0	-	-	-	20.4	75.3	/	[34]
66.12	15	7.16	2.57	1.19	0.26	0.54	3.52	1.14	9	88.28	N	[35]
80.8	5.1	1.6	3.1	-	1.5	-	6.3	0.8	0.4	87.5	F	[4]
58.6	9	8.4	4.6	1.6	1.9	-	5.4	-	6.5	76	N	[36]
88.2	2.3	5.1	0.6	0.4	<0.1	0.1	1.3	0.4	0.35	95.6	F	[10]
69.2	0.2	1.7	0.1	<0.1	0.1	-	0.3	0.1	1.04	71.1	F	[10]

**Table 2:** SCBA impacts on concrete properties.

Concrete Properties		SCBA Influences	Ref.
Physical properties	Hydration heat	Total heat and peak heat rate decline with SCBA replacement level increase	[32,34]
	Drying shrinkage	No consistent conclusion	[32,52]
	Surface resistivity	Increase with SCBA replacement level increase	[32]
	Setting time		[21,57,58]
	The water requirement for normal consistency of cement paste		[21,57]
	Workability		[59,60,61]
	Soundness	Small expansion, but less than permissible limit	[30]
Mechanical strength and microstructure	Compressive strength	Increases first and then decreases with SCBA replacement level increase	[21,32,34]
		Decrease with incorporated SCBA	[58]
	Split tensile strength	Increases first and then decreases with replacement rate increase	[21,62]
	Flexural strength	Increases with SCBA content and curing age increase	[52,60]
	Modulus of elasticity	Almost unchanged	[63]
		Decreases with replacement rate increase	[60]
	Interfacial transition zone (ITZ)	Thickness reduces, indentation modulus and hardness increase	[27,59]
	Porosity	Increases with replacement rate increase	[58,64]
Durability	Chloride penetration	Significant decrease	[32,63,64]
	Chloride diffusion		[21,64]
	Gas penetration	Significant increase	[32]
	Coefficient of water absorption	Decreases first and increases with SCBA replacement level increase	[21,64]
	Sulfate resistance	Improves at certain SCBA replacements	[24]
	Resistance to elevated temperatures	Resistance improves with the incorporation of SCBA	[65]
	Alkali-silicon reaction	No research found	
	Freezing-thawing cycle		
	Dry and wet cycles		

**Table 3:** Optimal SCBA replacement for different concrete properties [28].

Property	Remark Range
Coefficient of water absorption	15%
Setting time	20%
Compressive strength	23–30%
Split tensile strength	5–20%
Flexural strength	15%
Modulus of elasticity	50%
Chloride penetration	25–30%
Chloride diffusion	25%
Soundness	25%

**Table 4:** Physical characteristics of burnt samples [30].

Characteristics	Raw SCBA	600 °C	700 °C	800 °C	900 °C
SAI at 7 days (%)	71	79	84	74	66
SAI at 28 days (%)	73	74	86	77	67
Loss on ignition (%)	21.0	16.0	14.0	12.8	8.0

## References

[1] Ambedkar B., Alex J., Dhanalakshmi J. (2017). Enhancement of mechanical properties and durability of the cement concrete by RHA as cement replacement: Experiments and modeling. Constr. Build. Mater..

[2] Thomas B.S., Kumar S., Arel H.S. (2017). Sustainable concrete containing palm oil fuel ash as a supplementary cementitious material—A review. Renew. Sustain. Energy Rev..

[3] Nakanishi E.Y., Frías M., Santos S.F., Rodrigues M.S., Villa R.V.D.L., Rodriguez O., Junior H.S. (2016). Investigating the possible usage of elephant grass ash to manufacture the eco-friendly binary cements. J. Clean Prod..

[4] Cordeiro G.C., Kurtis K.E. (2017). Effect of mechanical processing on sugar cane bagasse ash pozzolanicity. Cem. Concr. Res..

[5] Adesanya D.A., Raheem A.A. (2009). Development of corn cob ash blended cement. Constr. Build. Mater..

[6] Ban C.C., Ramli M. (2011). The implementation of wood waste ash as a partial cement replacement material in the production of structural grade concrete and mortar: An overview. Resour. Conserv. Recycl..

[7] Rodier L., Bilba K., Onesippe C., Arsene M.A. (2017). Study of pozzolanic activity of bamboo stem ashes for use as partial replacement of cement. Mater. Struct..

[8] Zhou S., Zhang X.A., Chen X. (2012). Pozzolanic activity of feedlot biomass (cattle manure) ash. Constr. Build. Mater..

[9] Fava G., Ruello M.L., Corinaldesi V. (2011). Paper mill sludge ash as supplementary cementitious material. J. Mater. Civ. Eng..

[10] Sales A., Lima S.A. (2010). Use of Brazilian sugarcane bagasse ash in concrete as sand replacement. Waste Manage..

[11] Deepika S., Anand G., Bahurudeen A., Santhanam M. (2017). Construction products with sugarcane bagasse ash binder. J. Mater. Civ. Eng..

[12] Teixeira S.R., Magalhaes R.S., Arenales A., Souza A.E., Romero M., Rincon J.M. (2014). Valorization of sugarcane bagasse ash: Producing glass-ceramic materials. J. Environ. Manage..

[13] Noorul A., Faisal M., Muhammad K., Gul S. (2016). Synthesis and characterization of geopolymer from bagasse bottom ash, waste of sugar industries and naturally available china clay. J. Clean Prod..

[14] Teixeira S.R., De Souza A.E., Santos G.T.D., Pena A.F.V., Miguel A.G. (2008). Sugarcane bagasse ash as a potential quartz replacement in red ceramic. J. Am. Ceram. Soc..

[15] Patcharin W., Sriamporn K., Kanokkan A., Fan W. (2012). Utilization biomass from bagasse ash for phillipsite zeolite synthesis. Manufacturing Science and Technology.

[16] Worathanakul P., Mothong P., Engkawara P. (2013). Fe_2_O_3_-SiO_2_ nanocomposite derived from bagasse ash for Cr(VI) removal. J. Biobased Mater. Bioenergy.

[17] Tchakoute H.K., Ruscher C.H., Hinsch M., Djobo J.N.Y., Kamseu E., Leonelli C. (2017). Utilization of sodium waterglass from sugar cane bagasse ash as a new alternative hardener for producing metakaolin-based geopolymer cement. Chem. Erde-Geochem..

[18] Nazriati N., Setyawan H., Affandi S., Yuwana M., Winardi S. (2014). Using bagasse ash as a silica source when preparing silica aerogels via ambient pressure drying. J. Non-Cryst. Solids.

[19] Rahman N.A., Widhiana I., Juliastuti R., Setyawan H. (2015). Synthesis of mesoporous silica with controlled pore structure from bagasse ash as a silica source. Colloid Surf. A.

[20] Hernández J.F.M., Middendorf B., Gehrke M., Budelmann H. (1998). Use of wastes of the sugar industry as pozzolana in lime-pozzolana binders: Study of the reaction. Cem. Concr. Res..

[21] Ganesan K., Rajagopal K., Thangavel K. (2007). Evaluation of bagasse ash as supplementary cementitious material. Cem. Concr. Compos..

[22] Chusilp N., Chai J., Kiattikomol K. (2009). Utilization of bagasse ash as a pozzolanic material in concrete. Constr. Build. Mater..

[23] Cordeiro G.C., Toledo R.D., Fairbairn E.D.R. (2008). Use of ultra-fine sugar cane bagasse ash as mineral admixture for concrete. ACI Mater. J..

[24] Joshaghani A., Ramezanianpour A.A., Rostami H. Effect of incorporating Sugarcane Bagasse Ash (SCBA) in mortar to examine durability of sulfate attack. Proceedings of the Second International Conference on Concrete Sustainability.

[25] Lima S.A., Sales A., Almeida F.D.C.R., Moretti J.P., Portella K.F. (2011). Concretes made with sugarcane bagasse ash: evaluation of the durability for carbonation and abrasion tests. Ambient Constr..

[26] Santos I., Rodrigues J.P.L., Ramos C.G., Martuscelli C.C., Castañon U.N., Alves V.C.C., Abreu G.M. (2017). Effect of the chemical attack on the properties of cimentititous composites with partial substitution of ash from sugar cane bagasse in natura. Matéria (Rio de Janeiro).

[27] Rossignolo J.A., Rodrigues M.S., Frias M., Santos S.F., Savastano H. (2017). Improved interfacial transition zone between aggregate-cementitious matrix by addition sugarcane industrial ash. Cem. Concr. Compos..

[28] Katare V.D., Madurwar M.V. (2017). Experimental characterization of sugarcane biomass ash—A review. Constr. Build. Mater..

[29] Frias M., Villar E., Savastano H. (2011). Brazilian sugar cane bagasse ashes from the cogeneration industry as active pozzolans for cement manufacture. Cem. Concr. Compos..

[30] Bahurudeen A., Santhanam M. (2015). Influence of different processing methods on the pozzolanic performance of sugarcane bagasse ash. Cem. Concr. Compos..

[31] Embong R., Shafiq N., Kusbiantoro A., Nuruddin M.F. (2016). Effectiveness of low-concentration acid and solar drying as pre-treatment features for producing pozzolanic sugarcane bagasse ash. J. Clean Prod..

[32] Bahurudeen A., Kanraj D., Dev V.G., Santhanam M. (2015). Performance evaluation of sugarcane bagasse ash blended cement in concrete. Cem. Concr. Compos..

[33] Batra V.S., Urbonaite S., Svensson G. (2008). Characterization of unburned carbon in bagasse fly ash. Fuel.

[34] Montakarntiwong K., Chusilp N., Tangchirapat W., Jaturapitakkul C. (2013). Strength and heat evolution of concretes containing bagasse ash from thermal power plants in sugar industry. Mater. Des..

[35] Ríos-Parada V., Jiménez-Quero V.G., Valdez-Tamez P.L., Montes-García P., Ríos-Parada V., Jiménez-Quero V.G., Valdez-Tamez P.L., Montes-García P., Ríos-Parada V., Jiménez-Quero V.G. (2017). Characterization and use of an untreated Mexican sugarcane bagasse ash as supplementary material for the preparation of ternary concretes. Constr. Build. Mater..

[36] Moraes J.C.B., Tashima M.M., Akasaki J.L., Melges J.L.P., Monzo J., Borrachero M.V., Soriano L., Paya J. (2016). Increasing the sustainability of alkali-activated binders: The use of sugar cane straw ash (SCSA). Constr. Build. Mater..

[37] Kamiya K., Ai O., Nasu H., Hashimoto T. (2000). Comparative study of structure of silica gels from different sources. J. Sol-Gel Sci. Technol..

[38] Cordeiro G.C., Tavares L.M., Toledo R.D. (2016). Improved pozzolanic activity of sugar cane bagasse ash by selective grinding and classification. Cem. Concr. Res..

[39] Cordeiro G.C., Toledo R.D., Tavares L.M., Fairbairn E.D.R. (2009). Ultrafine grinding of sugar cane bagasse ash for application as pozzolanic admixture in concrete. Cem. Concr. Res..

[40] Morales E.V., Villar-Cocina E., Frias M., Santos S.F., Savastano H. (2009). Effects of calcining conditions on the microstructure of sugar cane waste ashes (SCWA): Influence in the pozzolanic activation. Cem. Concr. Compos..

[41] de Soares M., Garcia D.C.S., Figueiredo R.B., Aguilar M.T.P., Cetlin P.R. (2016). Comparing the pozzolanic behavior of sugar cane bagasse ash to amorphous and crystalline SiO_2_. Cem. Concr. Compos..

[42] Arif E., Clark M.W., Lake N. (2016). Sugar cane bagasse ash from a high efficiency co-generation boiler: Applications in cement and mortar production. Constr. Build. Mater..

[43] Cordeiro G.C., Toledo R.D., Tavares L.M., Fairbairn E.M.R. (2012). Experimental characterization of binary and ternary blended-cement concretes containing ultrafine residual rice husk and sugar cane bagasse ashes. Constr. Build. Mater..

[44] Akram T., Memon S.A., Obaid H. (2009). Production of low cost self compacting concrete using bagasse ash. Constr. Build. Mater..

[45] Sua-Iam G., Makul N. (2013). Use of increasing amounts of bagasse ash waste to produce self-compacting concrete by adding limestone powder waste. J. Clean Prod..

[46] Somna R., Chai J., Rattanachu P., Chalee W. (2012). Effect of ground bagasse ash on mechanical and durability properties of recycled aggregate concrete. Mater. Des..

[47] Osinubi K.J., Bafyau V., Eberemu A.O. (2009). Bagasse Ash Stabilization of Lateritic Soil.

[48] Jamsawang P., Poorahong H., Yoobanpot N., Songpiriyakij S., Jongpradist P. (2017). Improvement of soft clay with cement and bagasse ash waste. Constr. Build. Mater..

[49] Khan S., Kamal M., Haroon M. (2015). Potential of cement-treated sugar cane bagasse ash (SCBA) as highway construction material. Road Transp. Res..

[50] Cordeiro G.C., Toledo R.D., Tavares L.M., Fairbairn E.M.R. (2008). Pozzolanic activity and filler effect of sugar cane bagasse ash in Portland cement and lime mortars. Cem. Concr. Compos..

[51] Castaldelli V.N., Akasaki J.L., Melges J.L.P., Tashima M.M., Soriano L., Borrachero M.V., Monzo J., Paya J. (2013). Use of slag/sugar cane bagasse ash (SCBA) blends in the production of alkali-activated materials. Materials.

[52] Arif E., Clark M.W., Lake N. (2017). Sugar cane bagasse ash from a high-efficiency co-generation boiler as filler in concrete. Constr. Build. Mater..

[53] Faria K.C.P., Gurgel R.F., Holanda J.N.F. (2012). Recycling of sugarcane bagasse ash waste in the production of clay bricks. J. Environ. Manage..

[54] Modani P.O., Vyawahare M.R. (2013). Utilization of bagasse ash as a partial replacement of fine aggregate in concrete. Procedia Eng..

[55] Moretti J.P., Sales A., Almeida F.C.R., Rezende M.A.M., Gromboni P.P. (2016). Joint use of construction waste (CW) and sugarcane bagasse ash sand (SBAS) in concrete. Constr. Build. Mater..

[56] Lura P., Wyrzykowski M., Tang C., Lehmann E. (2014). Internal curing with lightweight aggregate produced from biomass-derived waste. Cem. Concr. Res..

[57] Singh N.B., Singh V.D., Rai S. (2000). Hydration of bagasse ash-blended portland cement. Cem. Concr. Res..

[58] Tantawy M.A., El-Roudi A.M., Salem A.A. (2012). Immobilization of Cr(VI) in bagasse ash blended cement pastes. Constr. Build. Mater..

[59] Hussein A.A.E., Shafiq N., Nuruddin M.F., Memon F.A. (2014). Compressive strength and microstructure of sugar cane bagasse ash concrete. Res. J. Appl. Sci. Eng. Technol..

[60] Srinivasan R., Sathiya K. (2010). Experimental study on bagasse ash in concrete. Int. J. Serv. Learn. Eng..

[61] Patel J.A., Raijiwala D. (2015). Experimental study on compressive strength of concrete by partially replacement of cement with sugar cane bagasse ash. Int. J. Eng. Res. Appl..

[62] Amin N. (2011). Use of bagasse ash in cement and its Impact on the mechanical behavior and chloride resistivity of mortar. Adv. Cem. Res..

[63] Rerkpiboon A., Tangchirapat W., Jaturapitakkul C. (2015). Strength, chloride resistance, and expansion of concretes containing ground bagasse ash. Constr. Build. Mater..

[64] Rukzon S., Chindaprasirt P. (2012). Utilization of bagasse ash in high-strength concrete. Mater. Des..

[65] Gar P.S., Suresh N., Bindiganavile V. (2017). Sugar cane bagasse ash as a pozzolanic admixture in concrete for resistance to sustained elevated temperatures. Constr. Build. Mater..

[66] Hwang C.L., Shen D.H. (1991). The effects of blast-furnace slag and fly ash on the hydration of Portland cement. Cem. Concr. Res..

[67] Hossain K.M.A. (2004). Properties of volcanic pumice based cement and lightweight concrete. Cem. Concr. Res..

[68] Chusilp N., Chai J., Kiattikomol K. (2009). Effects of LOI of ground bagasse ash on the compressive strength and sulfate resistance of mortars. Constr. Build. Mater..

[69] Ganesan K., Rajagopal K., Thangavel K. (2007). Evaluation of bagasse ash as corrosion resisting admixture for carbon steel in concrete. Anti-Corros. Methods Mater..

[70] Bahurudeen A., Santhanam M. (2014). Performance evaluation of sugarcane bagasse ash-based cement for durable concrete. Emerg. Binder Mater..

[71] Shi C. (1998). Effect of supplementary cementing materials on the specific conductivity of pore solution and its implications on the rapid chloride permeability test (AASHTO T277and ASTM C1202) results. ACI Mater. J..

[72] Nair D.G., Fraaij A., Klaassen A.A.K., Kentgens A.P.M. (2008). A structural investigation relating to the pozzolanic activity of rice husk ashes. Cem. Concr. Res..

[73] Rashad A.M. (2013). Metakaolin as cementitious material: History, scours, production and composition—A comprehensive overview. Constr. Build. Mater..

[74] Cordeiro G.C., Toledo R.D., Fairbairn E.M.R. (2009). Effect of calcination temperature on the pozzolanic activity of sugar cane bagasse ash. Constr. Build. Mater..

[75] Cordeiro G.C., Barroso T.R., Filho R.D.T. (2018). Enhancement the properties of Sugar Cane Bagasse Ash with high carbon content by a controlled re-calcination process. KSCE J. Civ. Eng..

[76] Frias M., Villar-Cocina E., Valencia-Morales E. (2007). Characterisation of sugar cane straw waste as pozzolanic material for construction: Calcining temperature and kinetic parameters. Waste Manage..

[77] Bahurudeen A., Marckson A.V., Kishore A., Santhanam M. (2014). Development of sugarcane bagasse ash based Portland pozzolana cement and evaluation of compatibility with superplasticizers. Constr. Build. Mater..

